# Therapeutic Role of Repetitive Transcranial Magnetic Stimulation in Alzheimer’s and Parkinson’s Disease: Electroencephalography Microstate Correlates

**DOI:** 10.3389/fnins.2022.798558

**Published:** 2022-02-16

**Authors:** Lutfu Hanoglu, Eren Toplutas, Mevhibe Saricaoglu, Halil Aziz Velioglu, Sultan Yildiz, Burak Yulug

**Affiliations:** ^1^Department of Neurology, School of Medicine, Istanbul Medipol University, Istanbul, Turkey; ^2^Functional Imaging and Cognitive-Affective Neuroscience Lab (fINCAN), Health Sciences and Technology Research Institute (SABITA), Regenerative and Restorative Medicine Research Center (REMER), Istanbul Medipol University, Istanbul, Turkey; ^3^Program of Neuroscience Ph.D., Graduate School of Health Sciences, Istanbul Medipol University, Istanbul, Turkey; ^4^Program of Electroneurophysiology, Vocational School, Istanbul Medipol University, Istanbul, Turkey; ^5^Department of Women’s and Children’s Health, Karolinska Institutet, Stockholm, Sweden; ^6^Department of Neurology, School of Medicine, Alanya Alaaddin Keykubat University, Alanya, Turkey

**Keywords:** Alzheimer’s disease, Parkinson’s disease, EEG, rTMS, microstate analysis

## Abstract

**Introduction:**

The microstate analysis is a method to convert the electrical potentials on the multi-channel electrode array to topographical electroencephalography (EEG) data. Repetitive transcranial magnetic stimulation (rTMS) is a non-invasive method that can modulate brain networks. This study explores the pathophysiological changes through microstate analysis in two different neurodegenerative diseases, Alzheimer’s (AD) and Parkinson’s disease (PD), characterized by motor and cognitive symptoms and analysis the effect of rTMS on the impaired cognitive and motor functions.

**Materials and Methods:**

We included 18 AD, 8 PD patients, and 13 age-matched controls. For both groups, we applied 5 Hz rTMS on the left pre-SMA in PD patients while 20 Hz rTMS on the left lateral parietal region in AD patients. Each patient was re-evaluated 1 week after the end of the sessions, which included a detailed clinical evaluation and measurement of EEG microstates.

**Results:**

At the baseline, the common findings between our AD and PD patients were altered microstate (MS) B, MS D durations and transition frequencies between MS A–MS B, MS C–MS D while global explained variance (GEV) ratio and the extent and frequency of occurrence of MS A, MS B, and MS D were separately altered in AD patients. Although no specific microstate parameter adequately differentiated between AD and PD patients, we observed significant changes in MS B and MS D parameters in PD patients. Further, we observed that Mini-Mental State Examination (MMSE) performances were associated with the transition frequencies between MS A–MS B and MS C–MS D and GEV ratio. After left parietal rTMS application, we have observed significantly increased visual memory recognition and clock drawing scores after left parietal rTMS application associated with improved microstate conditions prominent, especially in the mean duration of MS C in AD patients. Also, pre-SMA rTMS resulted in significant improvement in motor scores and frequency of transitions from MS D to MS C in PD patients.

**Conclusion:**

This study shows that PD and AD can cause different and similar microstate changes that can be modulated through rTMS, suggesting the role of MS parameters and rTMS as a possible combination in monitoring the treatment effect in neurodegenerative diseases.

## Introduction

Neurodegenerative diseases are devastating conditions with progressive pathologies affecting neurons in both the central and the peripheral nervous systems (Rekatsina et al., 2020). In many neurodegenerative diseases, including Alzheimer’s (AD) and Parkinson’s disease (PD), neuronal networks undergo complex progressive degeneration manifested by both morphological and functional modifications leading to gradual changes in cognitive, behavioral, and motor skill functions (Katsnelson et al., 2016). Neurodegenerative diseases are thought to share common pathophysiological mechanisms highlighted by the aggregation of misfolded proteins and neuroinflammation which leads to progressive central nervous system impairments. To date, there is no effective treatment for neurodegenerative diseases, that would prevent, halt or reverse the disease course. All pharmacological treatment options are therefore mere symptomatic treatments that alleviate but do not change the disease course.

Dementia is a large group of neurodegenerative diseases in which at least two of the following cognitive functions, such as memory, speech, perception, calculation, judgment, abstract thinking, and problem solving, must be impaired (Maresova et al., 2020). AD is the most common form, affecting 60–80% of those living with dementia (Calabrò et al., 2021). Yet, there has been sufficient evidence to show cognitive domains that are particularly impaired in patients with AD are memory, attention and executive functions (Spaan et al., 2005, 2010).

Parkinson’s disease, in contrast, is the most common neurodegenerative movement disorder characterized by motor symptoms, such as tremor, rigidity, bradykinesia, and postural instability, and is the second most common neurodegenerative disease worldwide (Miranda and Greenamyre, 2017). Although most patients with AD and PD benefit from drugs that transiently restore the neurotransmitter levels, these treatment options are insufficient to modify the neurodegenerative clinical courses.

Repetitive transcranial magnetic stimulation (rTMS) is a safe, non-invasive, and efficacious technique when targeting specific areas of cortical dysfunction in depression, and a similar approach yielded therapeutic benefits both in AD and PD if applied to relevant cortical regions (Wassermann and Lisanby, 2001; Kobayashi and Pascual-Leone, 2003; Cotelli et al., 2008). Several studies have reported 5 Hz rTMS therapy as an effective treatment for the control of motor symptoms in PD, especially when applied to the pre-supplementary motor region. Similarly, targeting the DLPFC and lateral parietal cortex with 20 Hz rTMS has been suggested as an effective tool in enhancing cognitive functions in AD (Chou et al., 2020; Hanoǧlu et al., 2020; Nardone et al., 2020).

Electroencephalography (EEG) is a useful method for the evaluation of cortical electrophysiology with high temporal resolution. There are various analytical approaches available to extract information from unstructured EEG signals. Microstate analysis is one of these methods that can deal with quasi-stable brief patterns of coordinated topographical electrical patterns that remain transiently stable for 80–120 ms before rapidly transitioning into a new topographical state (Lehmann et al., 2009). The four dominant classes of microstate (MS), categorized A, B, C, and D, are observed in resting-state EEG and can explain 70% of the global variance of the data (Michel and Koenig, 2018). These above-mentioned four temporal parameters of microstate conditions are used to quantify brain dynamic changes that can represent alterations in various neurological and psychiatric diseases related to different cognitive or behavioral states: these above-mentioned four temporal parameters of microstate conditions are used to quantify brain dynamic changes that can represent alterations in various neurological and psychiatric diseases related to different cognitive or behavioral states: MA A exhibits a left-right orientation, MS B presents a right-left orientation, MS C covers an anterior-posterior orientation, and MS D associate with central orientation (Michel and Koenig, 2018). A further advantage of this approach is that it can be used to evaluate complex brain network functions impaired in several neurological and psychiatric diseases. In this relation several studies have suggested that EEG microstates are strongly associated with neurophysiological correlates of Resting State Networks identified by functional magnetic resonance imaging (fMRI), suggesting that Resting State Networks can be represented both by fMRI and EEG microstates (Britz et al., 2010; Custo et al., 2017). In view of this, it is also reasonable to assume that microstate analysis can be used for monitoring the treatment response through evaluating electrophysiological alterations caused by the specific treatment modalities, such as rTMS, which has been already established to induce critical changes in the brain’s electrophysiological architecture. The primary goal of this study was to determine the microstate correlates of motor and behavioral symptoms in AD and PD and evaluate the therapeutical correlates of rTMS on cognitive, motor and neurophysiological changes through analyzing the EEG microstate analysis.

## Materials and Methods

### Participants

This study included 18 AD patients, 8 PD patients, and 13 age-matched healthy participants. According to the National Institute of Neurological and Communicative Disorders and Stroke and the Alzheimer’s Disease and Related Disorders Association, AD patients were given a clinical diagnosis of AD (NINCDS-ADRDA). According to the UK Brain Bank criteria, PD patients were diagnosed with idiopathic PD (Daniel and Lees, 1993). Inclusion criteria for AD patients were as follows: 1 or 2 stages according to the Clinical Dementia Scale, lack of neurological or psychiatric disease other than AD, no medication or dose change during treatment, absence of serious mental or psychological disorder. Inclusion criteria for PD patients were as follows: patients diagnosed with PD, no other neurological or psychiatric disease other than Parkinsonism, no medication or dose change during treatment, absence of serious mental or psychological disorder. Inclusion criteria for healthy participants were as follows: no neurological or psychiatric disease, no medication, and a Mini-Mental State Examination (MMSE) score of 24 or higher (Güngen et al., 2002). The exclusion criteria for AD and PD patients were due to TMS safety concerns, the presence of metal, implantable devices such as pacemakers, and the use of anti-epileptic drugs (Dvořák et al., 1991; Rossi et al., 2009). All of the participants were right-handed. All participants provided written informed consent to the study, which was approved by Istanbul Medipol University’s Local Ethics Committee (Ethical Report No: E-10840098-772.02-4598).

### Study Design

Electroencephalography recordings and neuropsychometric tests were administered to all participants. In addition, the motor scale of PD patients was evaluated. The UPDRS-Motor scores of PD patients were 18.38 ± 5.18 before rTMS sessions. In AD patients, 20 Hz rTMS was used on the left lateral parietal region, whereas 5 Hz rTMS was used on the left pre-SMA region in PD patients (Hanoǧlu et al., 2020; Velioglu et al., 2021). All patients received ten sessions of rTMS with an 8 coil of 70 mm diameter over 2 weeks. The duration of the rTMS treatment was 20–30 min. One week after the end of the sessions, all patients were re-evaluated.

### Neuropsychometric and Motor Evaluation

The MMSE was used to assess global cognition for all participants. Digit Span Test is used to assess attention function in all patients. Memory is assessed using the Weschler Memory Scale (WMS) Logical Memory (short- and long-term) and WMS Visual Memory (short- and long-term); language ability is assessed using the Boston Naming Test; visual and perceptual functions are assessed using the Judgment of the Line Orientation Test and the Benton Facial Recognition Test; and executive functions are assessed using the Stroop Test and the Clock Drawing (Karakas et al., 1996, 1999; Akça Kalem et al., 2005). The motor subdivision of the United Parkinson’s Disease Rating Scale (UPDRS-III) was used to assess PD patients’ motor symptoms (Akbostancı et al., 2000).

### Repetitive Transcranial Magnetic Stimulation Protocol

Repetitive transcranial magnetic stimulation was applied through a navigation system using MNI coordinates based on Talairach atlas. The Powermag 100 Research TMS was used in conjunction with the Power Mag CMS20 measuring system (Germany). All patients underwent brain magnetic resonance imaging in order to be uploaded to the Brainvoyager TMS Neuronavigation system. T1-weighted MR images with a resolution of 1 × 1 × 1 mm were obtained in 3T for TMS neuronavigation. The subject’s brain is registered to the MNI152 standard brain atlas, and the atlas’ regions are identified. Stimulation coordinates are then back-projected to native space for TMS neuronavigation.

Musculus Abductor Pollicis Brevis area was determined in the primary motor area with the created 3-D image in order to find motor threshold value before rTMS sessions. The Motor Evoked Potential protocol was repeated prior to each single ongoing therapy session. The resting EMG response of the Abductor Pollicis Brevis muscle was detected on the contralateral hand by giving progressively increased stimulus on top of the motor cortex. A response of 50 microvolts in at least 5 trials of 10 consecutive responses was accepted as a motor threshold. The rTMS was performed for 10 sessions using the Power Mag Stimulator, which was connected to a 70-mm diameter sized figure-8 coil. Prior to each TMS session, the patients were stimulated to the same point in all sessions by marking left lateral parietal cortex for AD patients and left pre-SMAs for PD patients on the T1-weighted MRI image of the TMS neuronavigation system. During rTMS, the center of the magnetic coil was positioned with the coil angled at 45° and only the edge of the coil resting on the scalp.

The protocol developed in the Velioglu et al. (2021) study for rTMS protocol and left lateral parietal cortex determination was used in this study. The MNI152 coordinates for the left lateral parietal cortex were *x* = −24, *y* = −18, and *z* = −18 (Wang et al., 2014). Over the course of 2 weeks, the Alzheimer’s patients received ten sessions of rTMS with an 8 coil 70 mm in diameter. Each session consisted of 1,640 × 2 continuous 20 Hz pulses.

The protocol developed in the Hanoǧlu et al. (2020) study was used in this study for rTMS protocol and determination of the left pre-SMA. In MNI152 coordinates, the left pre-SMA was *x* = −6, *y* = 9, and *z* = 60 (Tremblay and Gracco, 2009). Over the course of 2 weeks, the PD patients received ten sessions of rTMS with a 70-mm diameter 8 coil. In each session, 1,000 × 2 pulses at 5 Hz were delivered continuously.

### Electroencephalography Recording

A 4-min EEG was recorded from 30 scalp electrodes (FP1, FP2, F7, F3, Fz, F4, F8, FT7, FC3, FCz, FC4, FT8, T7, C3, Cz, C4, T8, TP7, CP3, CPz, CP4, TP8, P7, P3, Pz, P4, P8, O1, Oz, and O2) using the international 10–20 system with two linked ear references (A1 and A2). All data were collected from each subject using the BrainVision Recorder (Brain product, Munich, Germany) in a dimly lit, soundproof room in our electrophysiological research laboratory (REMER Clinical Electrophysiology and Neuro-modulation Research and Application Laboratory). EEG recordings were made with a low cut-off (s) of 1 and a high cut-off (Hz) of 100 Hz; sample rates of 500 Hz; and electrode impedances of 15 kΩ from 30 channels.

### Electroencephalography Preprocessing

For the dataset, 4 min of raw data with resting-state and eyes-closed conditions were collected. The EEGlab toolbox, which runs in the cross-platform MATLAB environment, pre-processes all datasets (Delorme and Makeig, 2004). The raw data was bandpass filtered between 2 and 20 Hz, and a 50 Hz notch filter was applied. Artifact subspace reconstruction was used to inspect all EEGs automatically (max acceptable 0.5-s window SD 10). Independent component analysis (ICA) was used to remove eye movements and muscle artifacts. Following that, EEGs were organized into 120-s artifact-free data for each participant.

### Electroencephalography Microstate Analysis

The microstate studies were carried out utilizing the Microstate analysis tool in MATLAB for EEGLAB (Version 1.2).^1^ Topographic maps of instantaneous maxima of Global Field Power were created. *K*-means clustering was used to construct the microstate maps of each participant. Previously, four ideally microstate class topographies were identified. Using the relevant literature, this study investigated the four types of microstate topography (Michel and Koenig, 2018).

The group clustering maps were calculated individually for the control, pre-rTMS, and post-rTMS groups (Pre-rTMS Alzheimer, Post-rTMS Alzheimer, Pre-rTMS Parkinson, Post-rTMS Parkinson, and Healthy Subject) using a permutation technique that is each microstate class from A to D (Koenig et al., 2002). The discovered healthy subject classes’ group clustering maps were utilized as templates to allocate separately microstate maps to patient groups.

The data was retrieved in order to calculate statistics for coverage (percent total time), occurrence (mean number of microstates per second), duration (mean duration of a microstate), and transition probability. The topographic analysis of variance (TANOVA) approach was also employed to assess the topographical differences across microstate class groupings (A, B, C, and D). This approach was implemented using the Ragu program.^2^

### Statistical Analysis

JAMOVI (Version 1.8.4.0) was utilized to do statistical analysis.^3^ To determine data normality, the Shapiro–Wilk normality test was used. For normally distributed data, parametric tests were used on all normally data, for the data is not normally distributed, non-parametric tests were used on all non-normally data. For comparison of microstate characteristics between control and rTMS groups, independent samples Student’s *t*-test and Mann–Whitney *U* were employed. For the comparison of before and post rTMS, paired samples Student’s *t*-test and Wilcoxon rank were employed. To compute correlations between data, Spearman’s correlation test was used. For all tests, the significance level was set at *p* < 0.05.

## Results

### Demographics and Clinical Features

There were no significant differences between the groups in terms of age, gender, or education (*p* > 0.05 independent samples Student’s *t*-test, Mann–Whitney *U* test and Chi-squared test). Table 1 shows the demographic statistics for both groups.

**TABLE 1:** Demographic data were given of AD, PD, and healthy control (HC) groups.

	AD (*n* = 18)	PD (*n* = 8)	HC (*n* = 13)	*p*-Value (AD/HC)	*p*-Value (PD/HC)
Age (mean ± SD)	70.67 ± 7.71	70.63 ± 4.63	69.15 ± 5.60	0.552	0.541
Sex (F/M)	12/6	1/7	5/8	0.110	0.201
Education (years) (mean ± SD)	7.83 ± 4.66	11.38 ± 4.69	9.08 ± 5.25	0.651	0.232

### Motor and Neuropsychometric Scores

Table 2 shows a comparison of motor and neuropsychometric scores. Neuropsychometric and motor test scores improved significantly after rTMS in both AD and PD groups as compared to baseline. The AD group scored better in the clock-drawing and visual memory recognition tasks after receiving rTMS (*p* = 0.031 and *p* = 0.048 respectively, paired samples Student’s *t*-test and Wilcoxon rank test). In the PD group, there was a substantial improvement in UPDRS-III scores following rTMS compared to baseline (*p* < 0.05, paired samples Student’s *t*-test).

**TABLE 2:** The assessments of motor and cognitive functions of health control (HC), AD, and PD groups at pre and post-rTMS.

	Pre-rTMS	Post-rTMS	Pre–post rTMS *p-*value	Pre-rTMS	Post-rTMS	Pre–post rTMS *p-*value	
**Group**	**AD**	**AD**	**AD**	**PD**	**PD**	**PD**	**HC**

*N*	18	18		8	8		13
MMSE (Mean ± SD)	17.93 ± 4.46	17.21 ± 3.62	0.522	24.13 ± 1.64	26.00 ± 3.07	0.110	27.46 ± 1.33
Clock drawing (Mean ± SD)	1.76 ± 1.68	2.35 ± 1.50	0.031*	1.50 ± 1.69	1.63 ± 1.77	0.773	−
UPDRS-III (Mean ± SD)	−	−	–	18.38 ± 5.18	13.88 ± 4.73	0.009*	−
Visual memory recognition (Mean ± SD)	1.28 ± 1.23	1.94 ± 1.55	0.048*	1.38 ± 1.51	1.75 ± 1.39	0.414	−

“*” represents significantly differences for p < 0.05.

### Microstate Topographies

**FIGURE 1:**
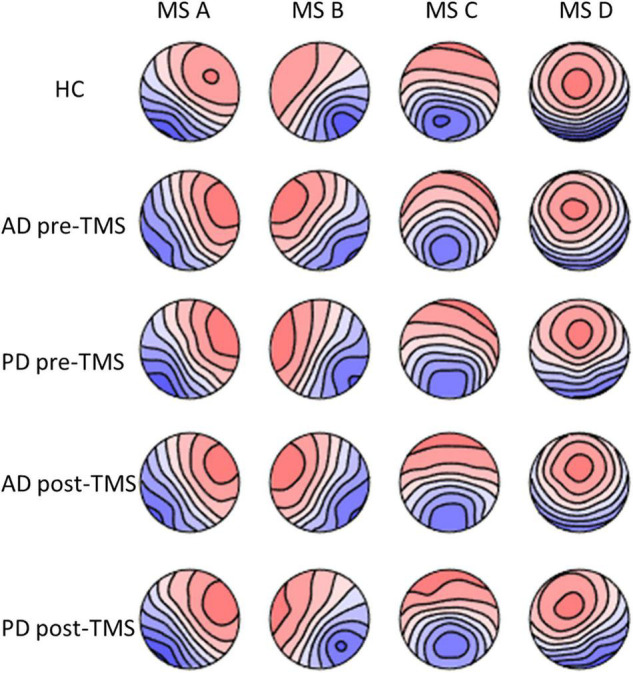
The microstate maps of PD and AD groups at pre-rTMS and post-rTMS and HC group. *p* > 0.05. MS A, microstate A; MS B, microstate B; MS C, microstate C; MS D, microstate D; HC, healthy control; AD, Alzheimer’s disease patients; PD, Parkinson’s disease patients; pre-rTMS, before rTMS sessions; post-rTMS, after rTMS sessions.

After comparing groups using a randomization test (TANOVA), there were no significant variations in terms of the individual microstate topographies of each microstate class. The topographies produced in healthy groups are comparable to those seen in the literature (Figure 1).

### Microstate Parameters

**FIGURE 2:**
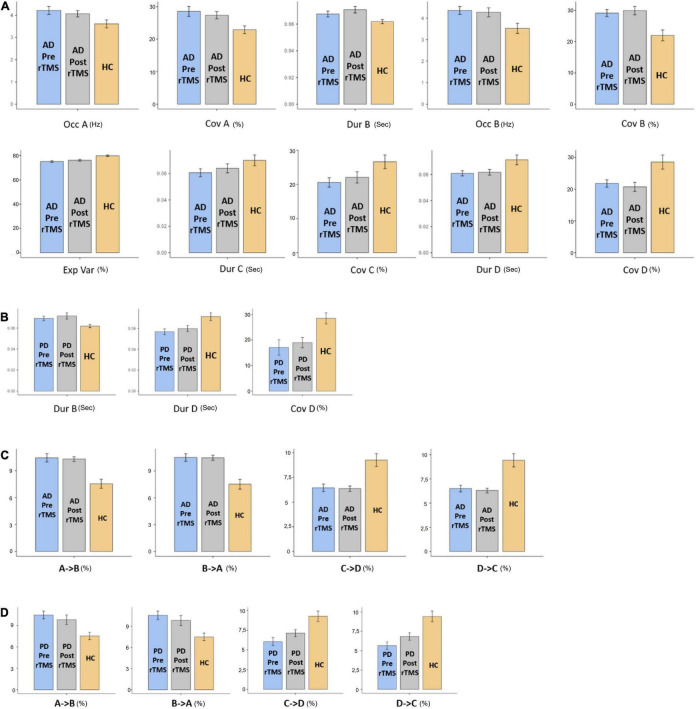
The statistics results of **(A)** AD and **(B)** PD groups of microstate characteristic as duration (Dur), explained variance (Exp Var), coverage (Cov) of microstate as A, B, C, and D transitions between microstates for **(C)** AD and **(D)** PD groups. “→” represents transitions between microstates.

The MS parameters of PD and AD patients were compared to those of a healthy control group (Tables 3, 4). The global explained variance (GEV) rate differed significantly between the Alzheimer and control groups (*p* = 0.001, independent samples Student’s *t*-test) (Figure 2). The GEV rate of Alzheimer’s patients was considerably enhanced by left lateral parietal cortex stimulation (*p* = 0.024, Wilcoxon rank test). When comparing the rate of occurrence and coverage of MS A (*p* = 0.028 and *p* = 0.010 respectively, independent samples Student’s *t*-test), there was a statistically significant higher in the AD group than in the control group. The mean duration of MS B in the AD group was longer (*p* = 0.042, Mann–Whitney *U* test), the occurrence of MS B was greater (*p* = 0.008, independent samples Student’s *t*-test), and the coverage rate was higher (*p* = 0.001, independent samples Student’s *t*-test) than in the control group for the MS B characteristics. There were no statistically significant changes in MS A and MS B metrics following rTMS in the AD group. The mean duration of MS C in the AD group was shorter (*p* = 0.028, Mann–Whitney *U* test), and coverage of MS C was lower (*p* = 0.015, independent samples Student’s *t*-test) than in the control group for the MS C parameters. After rTMS, the mean duration of MS C was prolonged in AD patients (*p* = 0.05, paired samples Student’s *t*-test). In terms of MS D characteristics, the AD group had a shorter mean duration (p = 0.014, independent samples Student’s *t*-test) and less coverage (*p* = 0.007, independent samples Student’s *t*-test). After rTMS, there was no substantial change in MS D parameters.

**TABLE 3:** Microstates parameters of AD, PD, and control groups at pre and post-rTMS.

	AD pre-rTMS	AD post-rTMS	PD pre-rTMS	PD post-rTMS	HC
Explained variance (%)	75.12 ± 2.924	76.14 ± 3.135	78.33 ± 7.106	78.96 ± 3.651	79.88 ± 2.237
Duration A (sec)	0.0681 ± 0.01193	0.0680 ± 0.01171	0.0704 ± 0.01579	0.0701 ± 0.00876	0.0636 ± 0.00605
Duration B (sec)	0.0675 ± 0.00917	0.0708 ± 0.01028	0.0692 ± 0.00604	0.0716 ± 0.00851	0.0618 ± 0.00561
Duration C (sec)	0.0606 ± 0.01267	0.0639 ± 0.01445	0.0809 ± 0.04441	0.0707 ± 0.01209	0.0699 ± 0.01456
Duration D (sec)	0.0609 ± 0.00841	0.0617 ± 0.00936	0.0568 ± 0.00777	0.0598 ± 0.00806	0.0712 ± 0.01339
Mean duration (sec)	0.0653 ± 0.00913	0.0673 ± 0.00953	0.0729 ± 0.01870	0.0691 ± 0.00719	0.0679 ± 0.00716
Occurrence A (Hz)	4.2149 ± 0.75384	4.0635 ± 0.61219	3.9390 ± 0.88483	3.8938 ± 0.49754	3.6102 ± 0.66404
Occurrence B (Hz)	4.3571 ± 0.77905	4.2695 ± 0.87752	4.0600 ± 1.21660	3.9217 ± 0.60340	3.5237 ± 0.84204
Occurrence C (Hz)	3.3886 ± 0.73243	3.4069 ± 0.62608	3.3605 ± 0.80669	3.6422 ± 0.49583	3.7692 ± 0.44690
Occurrence D (Hz)	3.6049 ± 0.75614	3.3862 ± 0.98489	3.0380 ± 1.60639	3.1454 ± 0.73895	3.9756 ± 0.70449
Overall occurrence (Hz)	15.5655 ± 1.92617	15.1261 ± 2.00146	14.3975 ± 3.13675	14.6030 ± 1.44018	14.8788 ± 1.52448
Coverage A (%)	28.52 ± 6.405	27.32 ± 4.710	27.40 ± 7.005	0.2720 ± 4.088	22.87 ± 4.293
Coverage B (%)	29.11 ± 4.722	29.89 ± 5.605	27.55 ± 6.480	0.2797 ± 5.018	21.97 ± 6.229
Coverage C (%)	20.58 ± 5.797	22.05 ± 6.982	27.97 ± 16.311	0.2583 ± 5.947	26.61 ± 7.143
Coverage D (%)	21.79 ± 5.004	20.74 ± 6.095	17.08 ± 8.568	0.1900 ± 5.787	28.55 ± 7.977

**TABLE 4:** The statistics results of the microstate parameters which are global explained variance (GEV), duration (Dur), occurrence (Occ), and coverage (Cov) in AD and PD groups.

	GEV	MS A			MS B			MS C			MS D		
		Dur	Occ	Cov	Dur	Occ	Cov	Dur	Occ	Cov	Dur	Occ	Cov
AD	↓		↑	↑	↑	↑	↑	↓		↓	↓		↓
PD					↑						↓		↓

*“↑” and “↓” represent increase and decrease, respectively. Note that there are microstate parameters before rTMS.*

**FIGURE 3:**
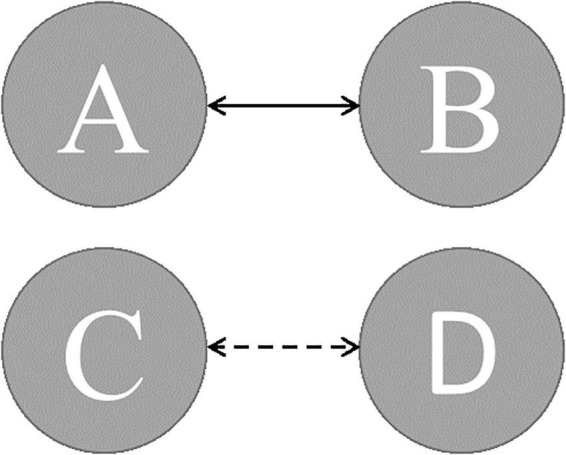
The expected transition probabilities in AD and PD patients (dashed line presents transition probabilities were lower; solid line presents transition probabilities were greater).

**FIGURE 4:**
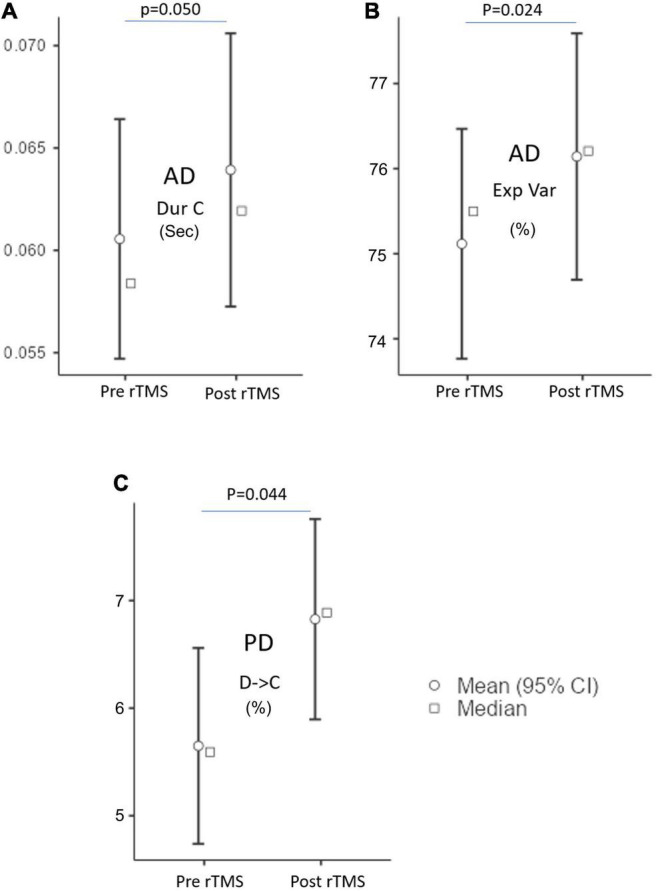
The microstate parameters which are **(A)** duration (Dur), **(B)** explained variance (Exp Var) of AD, and **(C)** transition of D to C of PD groups at pre-rTMS and post-rTMS. *p* < 0.05.

When compared to the mean duration of MS B, there was a statistically significant greater in the PD group than in the control group (*p* = 0.011, independent samples Student’s *t*-test). When compared to the mean duration of MS D, the PD group was statistically significantly shorter than the control group (*p* = 0.012, independent samples Student’s *t*-test). The MS D coverage rate in the PD group was lower than in the control group (*p* = 0.006, independent samples Student’s *t*-test) (Figures 3, 4). There were no statistically significant changes in MS parameters following rTMS in the PD group.

### The Probability of Expected Transition

The expected transition probabilities of the microstate topographies were compared to healthy controls in the AD and PD groups (Table 5). Expected transition probabilities from MS A to MS B and MS B to MS A were greater in AD, but transition probabilities from MS C to MS D and MS D to MS C were lower (*p* < 0.001, independent samples Student’s *t*-test). After rTMS, there was no significant change in the expected transition probabilities of AD patients. Similarly, in PD, the expected transition probability from MS A to MS B (*p* = 0.001, independent samples Student’s *t*-test) and MS B to MS A (*p* = 0.002, independent samples Student’s *t*-test) are greater, whereas the expected transition probabilities from MS C to MS D (*p* = 0.003, independent samples Student’s *t*-test) and MS D to MS C (*p* = 0.001, independent samples Student’s *t*-test) are lower. It was observed that after rTMS was administered to PD patients, the probability of expected transition from MS D to MS C increased (*p* = 0.044, paired samples Student’s *t*-test) (Table 6).

**TABLE 5:** Expected microstate transitions (%) of healthy control (HC), AD and PD groups at pre and post-TMS.

	AD pre-rTMS	AD post-rTMS	PD pre-rTMS	PD post-rTMS	HC
A→B	10.432 ± 1.972	10.290 ± 1.106	10.480 ± 1.526	9.820 ± 1.931	7.54 ± 1.81
A→C	8.164 ± 2.238	8.560 ± 2.823	9.295 ± 3.037	9.065 ± 0.916	8.17 ± 1.77
A→D	8.513 ± 1.431	8.062 ± 1.473	7.548 ± 2.467	7.815 ± 1.604	8.43 ± 1.16
B→A	10.509 ± 1.810	10.472 ± 1.259	10.575 ± 1.679	9.849 ± 1.999	7.51 ± 1.96
B→C	8.380 ± 1.416	8.799 ± 1.485	9.389 ± 3.083	9.228 ± 1.878	7.76 ± 1.32
B→D	9.025 ± 2.060	8.797 ± 2.455	7.890 ± 3.530	7.746 ± 0.922	8.20 ± 1.87
C→A	7.653 ± 2.061	8.208 ± 2.936	9.287 ± 3.618	8.888 ± 1.117	8.38 ± 2.01
C→B	7.822 ± 1.649	8.297 ± 1.749	9.388 ± 4.023	9.029 ± 1.999	7.98 ± 1.37
C→D	6.450 ± 1.593	6.374 ± 1.128	6.065 ± 1.423	7.126 ± 1.244	9.26 ± 2.36
D→A	8.074 ± 1.441	7.659 ± 1.809	7.086 ± 3.068	7.343 ± 1.757	8.79 ± 1.46
D→B	8.480 ± 2.047	8.183 ± 2.476	7.348 ± 3.904	7.264 ± 1.244	8.56 ± 1.83
D→C	6.499 ± 1.460	6.299 ± 1.003	5.649 ± 1.314	6.827 ± 1.344	9.42 ± 2.49

*Black arrows represents transitions between microstates.*

**TABLE 6:** The results of microstate parameters and clinical scales of rTMS of AD and PD groups.

rTMS protocol	Group	Microstate parameters	Clinical scales
Left lateral parietal 20 Hz 10 sessions	AD	Exp Var ↑ MS C Dur ↑	Clock drawing ↑ Visual memory recognition ↑
Left pre-SMA 5 Hz 10 sessions	PD	MS D → MS C The expected transition probabilities ↑	UPDRS-III ↑

*“↑” represents significantly increases. Parameters which are explained variance (Exp Var), duration (Dur), and transition of D to C (→).*

### Correlation Between Microstate Characteristics and Neuropsychological Evaluations

**FIGURE 5:**
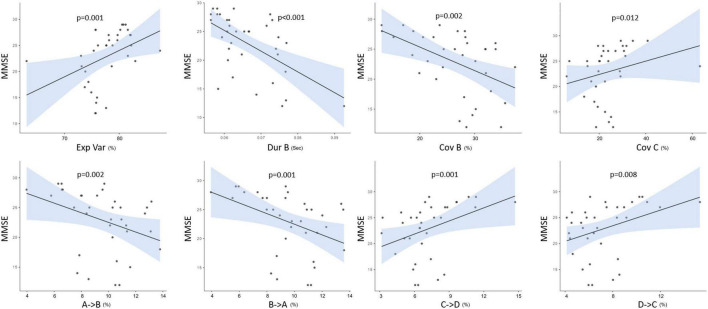
The correlations between MMSE and both microstate parameters as explained variance (Exp Var), duration (Dur), coverage (Cov), and the expected transition probabilities of all groups. *p* < 0.05.

Figure 5 illustrates a significant relationship between the MMSE scores of AD, PD, and healthy participants and different microstate parameters such as GEV, duration and coverage of MS B, and coverage of MS C. The similar relationship was seen in the expected transition probabilities of MS A to MS B, MS B to MS A, MS C to MS D, and MS D to MS C (Spearman’s correlation test). There was no significant connection between UPDRS motor scores and microstate characteristics in the PD group. Furthermore, no significant relationships were identified between the neuropsychological assessment test scores and microstate characteristics.

## Discussion

In our study, we observed significant differences in certain MS parameters between the neurodegenerative patient groups (AD and PD) and healthy controls. Despite some similarities, microstate parameters differed in many details between the AD and PD patients groups. We further observed that cognitive and motor scores were significantly improved after rTMS application in these patients. To evaluate the significance of these findings to the microstate correlates of the pathophysiological mechanisms, we also examined the microstate parameters before and after the rTMS application.

Common findings between our AD and PD patients were determined as the increasing duration in MS B, decreasing duration in MS D and increased frequency of transitions between MS A-MS B and decreased frequency of transitions between MS C-MS D. As reported here and previously by others for AD patients, we have found that increased MS B parameters were related to cognitive status also in PD patients (Smailovic et al., 2019; Chu et al., 2020; Lin et al., 2021). The initial research exploring EEG microstates in dementia patients found that microstate durations were shortening in these individuals (Dierks et al., 1997). However, as clustering analysis has progressed, more sophisticated approaches have emerged, and new conclusions have begun to emerge. Indeed, in our investigation, it was discovered that the durations of some particular microstates were prolonged and others were decreased, in accordance with current findings. Our observation was in agreement with recent PD data showing even decreased parameters of MS B values in PD patients after the anti-PD treatment (Serrano et al., 2018). These findings together might indicate common electrophysiological indicators of neurodegenerative symptomatology in different neurodegenerative disease conditions. Several resting-state network studies have reported a strong link between MS B status and the functional integrity of the visual network in healthy subjects involving the connections of the bilateral occipital cortex with other subcortical structures (Britz et al., 2010; Custo et al., 2017) shedding further light on the involvement of occipital regions in the neurodegenerative process. Our present findings of a negative correlation between MS B values and MMSE scores might therefore be of critical significance in understanding the indicator role of MS B in cognition.

Several studies suggested significant changes in MS D parameters and topographies in AD and PD patients, indicating a common network mechanism underlying the cognitive dysfunction (Serrano et al., 2018; Smailovic et al., 2019; Chu et al., 2020; Tait et al., 2020). However, a recent PD study reported significantly decreased parameters of MS D in PD patients even without dementia, although relevant topographical alterations were not found (Pal et al., 2020). Interestingly a recent meta-analysis covering a significant number of schizophrenia patients showed similarly decreased MS D values indicating a common dopaminergic dysfunction seen in both diseases (Rieger et al., 2016). Several fMRI studies found that MS D could be linked with the existing functionalities of frontoparietal, attention and executive networks, providing evidence for the role of MS D as a marker for impaired network activity in neurodegenerative diseases (Britz et al., 2010). These findings, along with the results obtained in the present study, make a “broader” predictive role of MS D in impaired network activity of non-motor symptoms in PD, including visual, perceptional and executive/memory networks, associated with the-of non-motor symptoms in PD. Other common findings in patients with AD and PD observed in the present study were increased frequency of transitions between MS A–MS B and decreased frequency of transitions between MS C–MS D. It has previously been observed that PD patients transition more frequent from MS B to MS A during off periods, whereas AD patients transition was less frequent from MS D to MS C and more from other microstates to MS A (Serrano et al., 2018; Musaeus et al., 2020). Transitions between microstates are considered to be non-random (Khanna et al., 2015). Interestingly, in evaluating the MMSE score correlation with the microstate parameters, we observed that MMSE performances were inversely and directly associated with frequency of transition between MS A–MS B and MS C–MS D, respectively, suggesting again a possible shared pathophysiologic mechanism between AD and PD. Beyond that, it is also reasonable to assume that increased frequency of transitions between MS A–MS B and decreased MS C–MS D are powerful indicators of improved and impaired cognitive performances related respectively to audiovisual and attention-salience network activity.

The findings mentioned above were also confirmed by evaluating microstate conditions specifically in separate AD and PD patients. For instance, especially in AD patients, we observed decreased GEV ratio, increased coverage and occurrence frequencies of MS A and B, decreased duration and coverage MS D. Here it is worth mentioning that although several studies with a healthy population have confirmed a variance ratio greater than 70%, our finding of 75% in AD patients is significantly lower than the healthy persons fitting well with the general concept of decreased brain oscillatory functions in AD (Khanna et al., 2015; Michel and Koenig, 2018). Accordingly, we determined a significant and direct correlation between MMSE scores and the GEV ratio in the present work. Likewise, our finding of increased and decreased microstate parameters (MS A, MS B, MS C, and MS D) agrees well with previous AD studies showing a similar level of alterations across the AD population (Musaeus et al., 2019, 2020; Smailovic et al., 2019). Beyond that, several studies indicated that microstates are associated with healthy and diseased network activity. For instance, Musaeus et al. (2019) have found that altered MS A activity is associated with temporal lobe dysfunction in AD and Mild Cognitive Impairment patients as suggested by its role in temporal lobe associated auditor network activity. Similarly, MS C has been found to reflect the saliency and cingulate gyrus network activity, hence, it is not surprising that FTD and AD patients showed decreased MS C parameters in previous studies (Nishida et al., 2013; Musaeus et al., 2019). As demonstrated here for MS C, our finding of a positive correlation between MMSE scores and MS C suggests MS C’s role in cognitive processes in this framework.

Although we observed significant alterations in MS B and MS D parameters in PD patients, we could not detect a specific microstates parameter that adequately distinguished between AD and PD patients. Furthermore, in contrast to previous studies showing decreased parameters of MS C in PD patients, we found no evidence of significant alterations in the MS C parameter (Chu et al., 2020; Pal et al., 2020). This was not surprising for us basically since MS C is being considered as a cognitive parameter, and our PD group showed no decline in cognitive parameters. This finding is in line with previous observations linking MS C with Montreal Cognitive Assessment scores and salience network activity in PD patients (Chu et al., 2020). A similar association for MS C has been also reported for MS A and Parkinsonian motor scores in recent studies (Chu et al., 2020). For instance, recent studies have found a significant correlation between UPDRS III scores and increased MS A, which was reversed with L-Dopa treatment (Serrano et al., 2018; Chu et al., 2020). Although rare reports suggest that evaluating multiple microstate parameters instead of one could better indicate an impaired motor function in PD, a fact could not be neglected that most of such existing studies are small sample-sized, and the role of MS A in neurodegeneration needs further studies. Nonetheless, it is quite interesting that the MS correlates well with critical disease parameters in our small study.

In evaluating the cognitive and motor responses and their relation with microstate parameters we have observed significantly increased visual memory recognition and clock drawing scores after left parietal rTMS application associated with improved microstate conditions prominent, especially in the mean duration of MS C. Our study’s improved MS C parameters fit well with recent studies in healthy persons and schizophrenia patients showing altered MS C values after rTMS left parietal application (Croce et al., 2018; Sverak et al., 2018). Despite these positive findings suggesting the critical role of MS C in cognitive impairment in AD, we have not observed a significant correlation between improved cognitive scores and MS C values which could be related to our small sample size. Due to the possibly same reason, we have not observed a significant correlation between microstate parameters, frequency of transitions from MS D to MS C, and UPDRS scores although pre-SMA rTMS resulted in significant improvement in motor scores and frequency of transitions from MS D to MS C which increased briteven to normal control levels in PD patients. Nevertheless, our present study might indicate that the transition rate from MS D to MS C may be a valuable predictor of PD-specific motor involvement. Although the current study contributes to the literature in several ways, certain limitations should be noted. First, our sample size was small, gender ratios were varied and included only a small number of patients which might be responsible for limited changes observed in microstate parameters and clinical scores. Second, we did not perform a longitudinal study, this study only examined the short-term effect of rTMS. Third, we did not stop current therapeutic regimens due to ethical reasons. Fourth, we assessed only the between 2 and 20 Hz frequency bands which were the most studied frequency ranges in recent MS studies. Microstate research in limited frequency ranges have revealed that microstates are the outcome of wider frequency band activities rather than being restricted to certain frequency ranges such as the alpha band (Croce et al., 2020). Although microstates are often researched throughout a wide variety of frequency bands, analyzing individual frequency ranges may also provide useful information. Therefore, confirmation of our results in randomized multiple cohorts by wider frequency range with also detailed analysis of delta, theta, alpha, and beta frequency bands would be of great interest.

## Conclusion

In conclusion, the microstates analysis method seems convenient and desirable to obtain dynamic and electrophysiological changes in neurodegenerative diseases. While specifically altered microstate transitions (such as between MS A–MS B and between MS C–MS D) were essential indicators of neurodegeneration and motor involvement during such a process, isolated microstate parameter changes also seemed to associate with the dysfunction of executive and memory functions: Therefore this study highlights that PD and AD may have different microstate parameters related to different disease-specific symptoms and suggests that a detailed microstate analysis can be a potential tool to identify neurophysiological disorders in neurodegenerative diseases. Microstate analysis seems to be indeed a very sophisticated method to monitor the effects of new neuromodulation methods such as rTMS, which have great potential to interfere with the functional state of the brain, as shown in the present work.

## Data Availability Statement

The raw data supporting the conclusions of this article will be made available by the authors, without undue reservation.

## Ethics Statement

The studies involving human participants were reviewed and approved by the Istanbul Medipol University’s Local Ethics Committee. The patients/participants provided their written informed consent to participate in this study.

## Author Contributions

ET, LH, and MS contributed to conception and design of the study. MS and HV organized the database. ET performed the statistical analysis. ET and MS wrote the first draft of the manuscript. LH, ET, MS, SY, and BY wrote sections of the manuscript. All authors contributed to manuscript revision, read, and approved the submitted version.

## Conflict of Interest

The authors declare that the research was conducted in the absence of any commercial or financial relationships that could be construed as a potential conflict of interest.

## Publisher’s Note

All claims expressed in this article are solely those of the authors and do not necessarily represent those of their affiliated organizations, or those of the publisher, the editors and the reviewers. Any product that may be evaluated in this article, or claim that may be made by its manufacturer, is not guaranteed or endorsed by the publisher.

## Footnotes

1
http://www.thomaskoenig.ch/Download/EEGLAB_Microstates

2
https://www.thomaskoenig.ch/index.php/software/ragu/download

3
https://www.jamovi.org

## Abbreviations

AD: Alzheimer’s disease
PD: Parkinson’s disease
rTMS: repetitive transcranial magnetic stimulation
EEG: electroencephalography
fMRI: functional magnetic resonance imaging
MS A: microstate A
MS B: microstate B
MS C: microstate C
MS D: microstate D
GEV: global explained variance
MMSE: Mini-Mental State Examination
WMS: Weschler Memory Scale.
